# Lifting Wavelet Transform De-noising for Model Optimization of Vis-NIR Spectroscopy to Predict Wood Tracheid Length in Trees

**DOI:** 10.3390/s18124306

**Published:** 2018-12-06

**Authors:** Ying Li, Brian K. Via, Qingzheng Cheng, Yaoxiang Li

**Affiliations:** 1 College of Engineering and Technology, Northeast Forestry University, Harbin 150040, China; yingli@nefu.edu.cn; 2 Forest Products Development Center, SFWS, Auburn University, Auburn, AL 36849, USA; brianvia@auburn.edu (B.K.V.); qzc0007@auburn.edu (Q.C.)

**Keywords:** lifting wavelet transform, Vis-NIR spectroscopy, larch, tracheid length

## Abstract

The data analysis of visible-near infrared (Vis-NIR) spectroscopy is critical for precise information extraction and prediction of fiber morphology. The objectives of this study were to discuss the de-noising of Vis-NIR spectra, taken from wood, to improve the prediction accuracy of tracheid length in Dahurian larch wood. Methods based on lifting wavelet transform (LWT) and local correlation maximization (LCM) algorithms were developed for optimal de-noising parameters and partial least squares (PLS) was employed as the prediction method. The results showed that: (1) The values of tracheid length in the study were generally high and had a great positive linear correlation with annual rings (R = 0.881), (2) the optimal de-noising parameters for larch wood based Vis-NIR spectra were Daubechies-2 (db2) mother wavelet with 4 decomposition levels while using a global fixed hard threshold based on LWT, and (3) the Vis-NIR model based on the optimal LWT de-noising parameters ($R_{c}^{2}$ = 0.834, RMSEC = 0.262, ${\mathrm{RPD}}_{c}$ = 2.454) outperformed those based on the LWT coupled with LCM algorithm (LWT-LCM) ($R_{c}^{2}$ = 0.816, RMSEC = 0.276, ${\mathrm{RPD}}_{c}$ = 2.331) and raw spectra ($R_{c}^{2}$ = 0.822, RMSEC = 0.271, ${\mathrm{RPD}}_{c}$ = 2.370). Thus, the selection of appropriate LWT de-noising parameters could aid in extracting a useful signal for better prediction accuracy of tracheid length.

## 1. Introduction

Visible and near-infrared (Vis-NIR) spectroscopy has been widely applied to agriculture, life science, polymer materials, and other fields [1,2,3] because of its many advantages, including simplicity of operation, no need for sample pretreatment, real-time online monitoring, and great adaptability of the model. It has been shown that Vis-NIR spectroscopy can be used in wood science for the prediction of wood’s physical, chemical, anatomical, and mechanical properties such as moisture content, microfibril angle, bending strength, etc. [4,5,6,7].

As an indirect and nondestructive method, Vis-NIR spectroscopy has the main bands of spectra that correspond to the stretching vibrations of C-H, N-H, O-H, and S-H in almost all biological samples [8]. Therefore, Vis-NIR could well characterize the major properties or compositions of the corresponding samples. Prediction of samples’ properties using Vis-NIR spectroscopy depends on chemometric analysis that explains the relationship between spectra and the corresponding properties. Both linear and nonlinear methods are used successfully for modeling algorithm, such as partial least squares (PLS) regression, support vector machine (SVM), least squares-support vector machine (LS-SVM), and artificial neural network (ANN) [9,10].

Wood, a complex natural organic compound, is mainly composed of cellulose, hemicellulose, and lignin. Wood microstructure parameters mainly include the microfibril angle, fiber length, tracheid length, crystallinity, or any fiber morphology that influence the wood physical, chemical, or mechanical properties [11]. These parameters are of importance for the forest genetics, trees-oriented breeding or the processing and use of wood. However, compared to the traditional methods of physical and chemical analysis, the determination of anatomical features is more complex, time-consuming, and places higher requirements upon forestry researchers. These determination methods cannot be used to realize the assessment of wood traits on a large-scale, multispecies and multisite.

Vis-NIR spectroscopy presents wood composition information through the interaction between Vis-NIR light and internal molecules. The goal for the prediction of wood properties is to develop a calibration model that relates Vis-NIR spectra to cell wall composition and morphology with multivariate statistics. Therefore, spectral quality is an important factor affecting the prediction precision for Vis-NIR models. However, environmental factors, spectral devices, surface roughness, and other various factors induce noise in spectra during acquisition [12,13], such as optical noise, electrical noise, and their interactions, which reduce prediction accuracy and precision. Determining how to remove noise while retaining as much useful information as possible is a challenge for forest researchers.

In recent years, spectral pretreatments including derivatives, multiplication scatter correction (MSC), standard normalized variate (SNV), Savitzky-Golay (SG) smoothing and wavelet transform (WT) [14,15,16] have been used widely for removing noise or other irrelevant information from the signal to improve model performance. While WT is superior in de-noising and compressing Vis-NIR data, removing the noise without losing useful information is still a challenge because the noise in the Vis-NIR spectra is mostly a random signal. Additionally, the traditional WT is time-consuming, and sometimes a large amount of calculations with advanced software is needed. In light of the above deficiencies, as the second-generation wavelet transform, lifting wavelet transform (LWT), with its advantages of rapid computing speed, simple algorithms, and lack of dependence on Fourier transform can be directly refactored into traditional wavelets in time domain and overcome the weakness of the boundary problem [17].

Dahurian larch is one of the major commercial tree species in northeastern China. Tracheid length could be a good indication for pulping properties and tensile strength of wood. Hence, the rapid determination of tracheid length is the key for timber quality classification and evaluation for fiber. In this study, Dahurian larch wood samples were used for Vis-NIR spectra collection and tracheid length estimation. Spectral data were processed by lifting wavelet transform (LWT) and the combination of local correlation maximization (LCM) algorithm. Wavelet transform (WT), moving average, loess, Savitzky-Golay and lowess were compared in this study, as well. The preprocessed Vis-NIR data were used for model development with the PLS method, which provides a theoretical basis and technical support for the rapid and accurate de-noising of wood Vis-NIR spectra.

## 2. Materials and Methods

### 2.1. Sample Preparation

**Figure 1:**
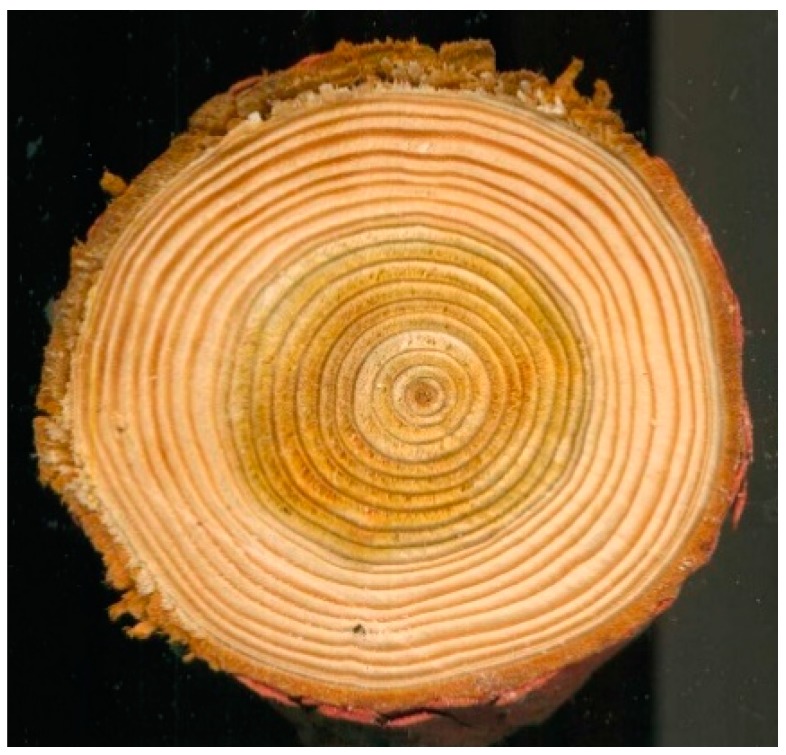
Dahurian larch disc sample.

Seven Dahurian larch (*Larix gmelinii*) trees ranging between 39 and 43 years in age were harvested from a larch plantation located at 45°44′00″–45°53′02″ N and 131°8′40″–131°21′23″ E, in Heilongjiang Province, China. Tree heights ranged from 18.8 to 22.5 m, with an average of 21.0 m and the crown width ranged from 2.8 to 4.6 m. The trees were removed from different parts to better represent the range of natural variability in the region. Five-centimeter-thick (5 cm) disks were removed from each tree near breast height (1.3 m) with a total of seven discs (Figure 1) used for model calibration. The disks were left to air dry in an environmental-controlled laboratory for three months. After removing bark from the edge of each disk, slab samples from pith to bark 2 × 2 × 5 cm^3^ in tangential, radial, and longitudinal dimensions were prepared for Vis-NIR spectra collection and tracheid length measurement with a total of 83 slab samples.

### 2.2. Vis-NIR Spectroscopy

The Vis-NIR spectra were collected from the rings of each sample using a LabSpec Pro FR/A114260 (Analytical Spectral Devices, Inc., Boulder, CO, USA). Thirty scans were obtained at 1 nm intervals, averaged into one spectrum, and saved as log (1/R). Because the ring width in the mature wood zone was smaller than the spot size of the fiber-optic probe, which was approximately 5 mm, the spectra from latewood portion were obtained [18]. Additionally, to reduce the noise from the spectrometer system, spectra data were collected for each ring from the 1st to 20th annual ring and in two ring intervals after the 21st annual ring. Wavelength range was limited to 350–2397 nm (wavelengths = 2048).

### 2.3. Tracheid Length Measurement and Calibrations

After Vis-NIR spectra were collected, the tracheid lengths of the latewood were detected by the isolation method [19], macerated with Jeffrey’s solution, and measured using a universal projector. Each tracheid length was recorded by averaging 25 measurements per sample.

The partial least squares (PLS) regression was conducted in the multivariate statistical analysis software called Unscrambler V10.4 (CAMO Software AS, Oslo, Norway). The models’ performance was assessed with the following prediction diagnostics [20], including the coefficient of determination (R^2^), root mean square error (RMSE), mean absolute percentage error (MAPE), standard error of calibration in the laboratory (SEC), and ratio of performance to standard deviation (RPD). The computation equations of these criteria are shown as follows:

(1) $$R^{2}=1-\sum_{i=1}^{n}{\left(y_{i}-\hat{y_{i}}\right)}^{2}/\sum_{i=1}^{n}{\left(y_{i}-\bar{y}\right)}^{2}$$

(2) $$\mathrm{RMSE}=\sqrt{\sum_{i=1}^{n}{\left(y_{i}-\hat{y_{i}}\right)}^{2}/n}$$

(3) $$\mathrm{MAPE}=\frac{1}{n}\sum_{i=1}^{n}\left|(y_{i}-\hat{y_{i}})/y_{i}\right|\times 100\%$$

(4) $$\mathrm{SEC}=\sqrt{\sum_{i=1}^{n}{\left(y_{i}-\hat{y_{i}}-\frac{1}{n}\sum_{i=1}^{n}(y_{i}-\hat{y_{i}})\right)}^{2}/n-1}$$

(5) $$\mathrm{RPD}=\mathrm{SD}/\sqrt{\sum_{i=1}^{n}{\left(y_{i}-\hat{y_{i}}-\frac{1}{n}\sum_{i=1}^{n}(y_{i}-\hat{y_{i}})\right)}^{2}/n-1}$$

where $y_{i}$ represents the tracheid length value, $\hat{y_{i}}$ and $\bar{y}$ are the predicted value and the mean of $y_{i}$, respectively. n is the number of samples. When n is the number of samples of the calibration set, coefficient of determination, ratio of performance to standard deviation and error are named $R_{c}^{2}$, ${\mathrm{RPD}}_{c}$, RMSEC and ${\mathrm{MAPE}}_{c}$, respectively. When n is the number of samples of the prediction set, coefficient of determination, ratio of performance to standard deviation and error are named $R_{p}^{2}$, ${\mathrm{RPD}}_{p}$ and RMSEP, respectively.

### 2.4. Vis-NIR Data Processing

#### 2.4.1. Lifting Wavelet Transform Analysis

The lifting wavelet transform (LWT), a second-generation wavelet, inherits the advantages of the multi-resolution of the traditional wavelet transform and has been recognized as a powerful signal processing tool with fast speed and small memory requirements. The basic steps of LWT are split, predict, and update [21,22]. The aim of LWT is to process lifting transform coefficients obtained by the decomposition of spectra based on the lifting scheme using the fixed threshold and then the reconstruction of the Vis-NIR spectra.

In this study, in order to ensure optimal de-noising parameters, four different mother wavelets (symN, biorN, rbioN, and dbN) were first used with the hypotheses of 5 decomposition layer (k = 5) and order (N) of 5 (N = 5.5 for bior and rbio wavelet) for the optimal wavelet, and then the spectral data were pretreated with a different order. After the optimal order was determined, the processed spectra were analyzed by different decomposition levels.

The LWT was implemented using MatlabR2014b (MathWorks, Natick, MA, USA), and the $R_{c}^{2}$, RMSEC, and ${\mathrm{MAPE}}_{c}$ were used for measuring the optimal de-noising parameters including mother wavelet, decomposition layer (k), and order (N). Generally, higher R^2^ and lower RMSE and MAPE indicate a better prediction result [23].

#### 2.4.2. Local Correlation Maximization Algorithm

In view of the phenomenon that the noise removal of some wavelengths is insufficient or eliminates useful signals, the local correlation maximization (LCM) algorithm was operated under the optimal LWT de-noising parameters. It was used for construction of the spectra through selecting the absorbance with the highest correlation between decomposition levels (one to the optimal level) and tracheid length in the wavelength range of 350–2397 nm. The specific steps are as follows [24], and it was implemented by using Matlab R2014b.

- Correlation analysis: the correlation coefficient (r) between Vis-NIR spectra under different decomposition levels and tracheid length were obtained.
- Judgment analysis: for the wavelength range of 350–2397 nm, each wavelength corresponds to multiple correlation coefficients, and the decomposition layer with the largest r in all decomposition layers was selected as the decomposition layer for this wavelength.
- Construction of spectra: the spectra were constructed by the absorbance of the decomposition level with the largest r.

#### 2.4.3. Comparison with Basic De-Noising Methods

Environmental factors, spectrometer, human operation, and other various factors induce noise in Vis-NIR spectra, such as baseline drift and light scattering. To eliminate the influence of this noise and analyze the effect of LWT and LCM, wavelet transform (WT), moving average, loess, Savitzky-Golay and lowess were compared. The parameters of wavelet de-noising were selected according to the optimal parameters of LWT. Additionally, the segment size for moving average, loess, Savitzky-Golay, and lowess were discussed.

### 2.5. Overview of Vis-NIR Spectra De-Noising Processing

**Figure 2:**
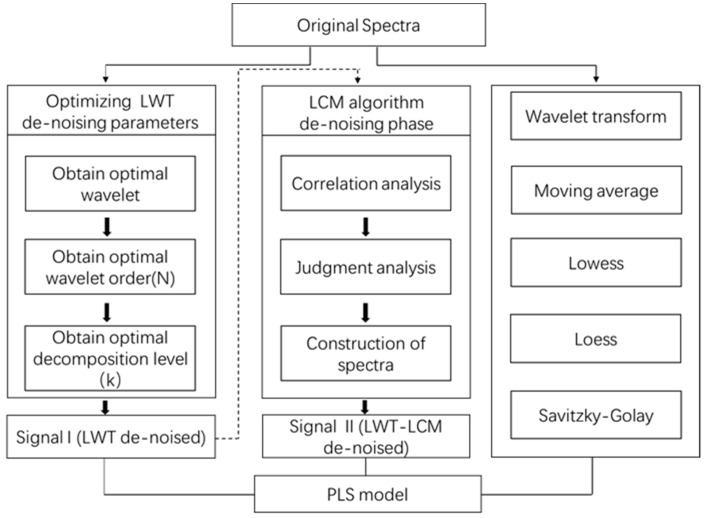
An overview of steps in Vis-NIR spectra processing.

A total of 164 samples were analyzed, which included a calibration set with 117 samples and a validation set with 47 samples. Firstly, Vis-NIR spectra of the calibration set were pretreated by four mother wavelets (sym5, bior5.5, rbio5.5 and db5) with five decomposition levels to ensure the optimal wavelet. The optimal order (N) and decomposition level (k) were then determined under the optimal wavelet. After the optimal LWT de-noising parameters were obtained, LCM algorithm, wavelet transform (WT), moving average, loess, Savitzky-Golay, and lowess were used for further de-noising. Finally, the processed spectral data were modeled by PLS. The total treatment is depicted in Figure 2.

## 3. Results and Analysis

### 3.1. Statistical Characteristics of Wood Tracheid Length

The total samples were randomly divided into a calibration set (117 samples) and a prediction set (47 samples). As illustrated in Table 1, the tracheid length ranged from 1.626 mm to 4.618 mm, with an average value of 3.320 mm, indicating that the larch tracheid length in this area was generally large relative to the literature. The distribution exhibited low negative skewness and kurtosis, indicating low scatter distribution.

### 3.2. Radial Development of Wood Tracheid Length in Annual Rings

**Figure 3:**
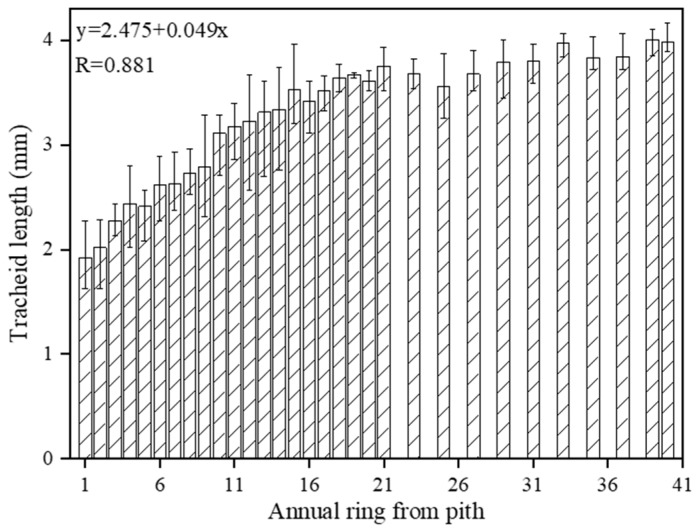
Radial variation in larch tracheid length. Data are averages (±SD) for the ring from 1 to 40.

A strong positive linear correlation between tracheid length and annual ring was found as shown in Figure 3, and the correlation coefficient was larger than 0.80. The tracheid length near the pith was relatively small and increased rapidly before 18 years and levelled off between the 23rd and 35th annual rings. The tracheid length reached the maximum value at the 40 annual rings.

### 3.3. Selection of Optimal LWT De-Noising Parameters

As the first step of LWT de-noising, the choice of mother wavelets greatly influences the effects of de-noising, but there is no definitive guideline on how to select appropriate wavelets, so flexibility in selecting parameters is needed. In this study, symN, biorN, rbioN, and dbN wavelets were analyzed, and the assumed decomposition levels and order are all 5 (k = N = 5) under the global fixed threshold.

As shown in Table 2, the modeling results from bior5.5 and rbio5.5 were inferior to those from db5 and sym5, while the spectral data processed by db5 with the fifth decomposition level under the global fixed hard threshold had a higher accuracy, which may be due to the property of the biorthogonal compactly supported for the db wavelet [25]. Thus, the Vis-NIR spectra pretreated by the dbN wavelet family with fifth decomposition level under the global fixed hard threshold were used for selecting optimal order (N).

It is difficult to apply the dbN wavelet family without selecting an appropriate choice of order. To effectively remove noise, the effect of de-noising of different orders, (N = 1–8) for the db wavelet, are demonstrated in Table 3, and it can be seen that the results were influenced by wavelet orders. Among these models with dbN preprocessing, the performance of the db8 wavelet was the worst for the data set in which the $R_{c}^{2}$ was less than 0.50. The model with the db2 wavelet obtained the best results with a higher $R_{c}^{2}$ and lower RMSEC and ${\mathrm{MAPE}}_{c}$ ($R_{c}^{2}$ = 0.818, RMSEC = 0.274, ${\mathrm{MAPE}}_{c}$ = 7.443). Therefore, the optimal order for Vis-NIR spectra of larch wood is 2 (N = 2), and the spectra pretreated with db2 was used for further analysis.

It is a challenge to select appropriate decomposition levels for wavelet de-noising, but the levels between second to fifth were randomly selected in most studies [26]. After the optimal wavelet and order were selected, decomposition levels from first to fifth were analyzed.

**Figure 4:**
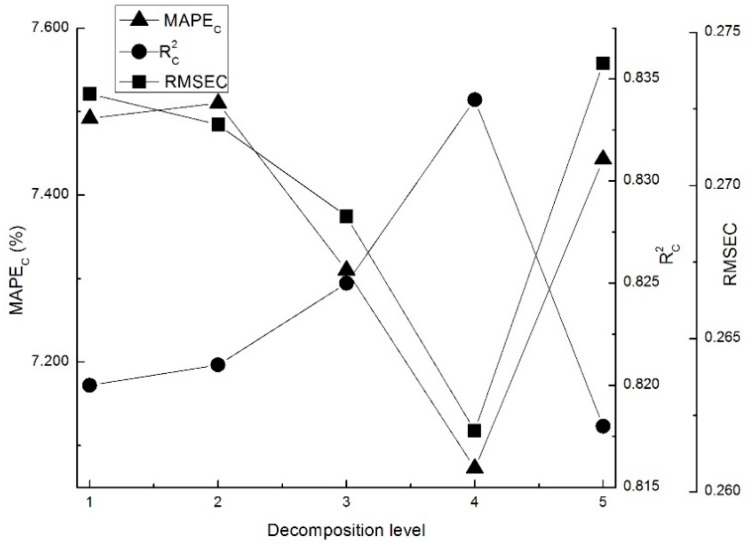
Results of PLS model for various decomposition levels of db2 wavelet.

As shown in Figure 4, the $R_{c}^{2}$ increased gradually first and then declined with increasing decomposition levels, and similar conclusions were also deduced by Cai [27] in the study on soil moisture content (SMC). Among models with first to fifth levels of the db2 wavelet, the optimal decomposition level is fourth because the $R_{c}^{2}$ was relatively higher than that of other models indicating that the noise or irrelative signal was removed. Overall, the optimal de-noising parameters of LWT for larch Vis-NIR spectra is db2 with fourth level (k = 4, N = 2).

### 3.4. Comparison with Basic De-Noising Methods

**Figure 5:**
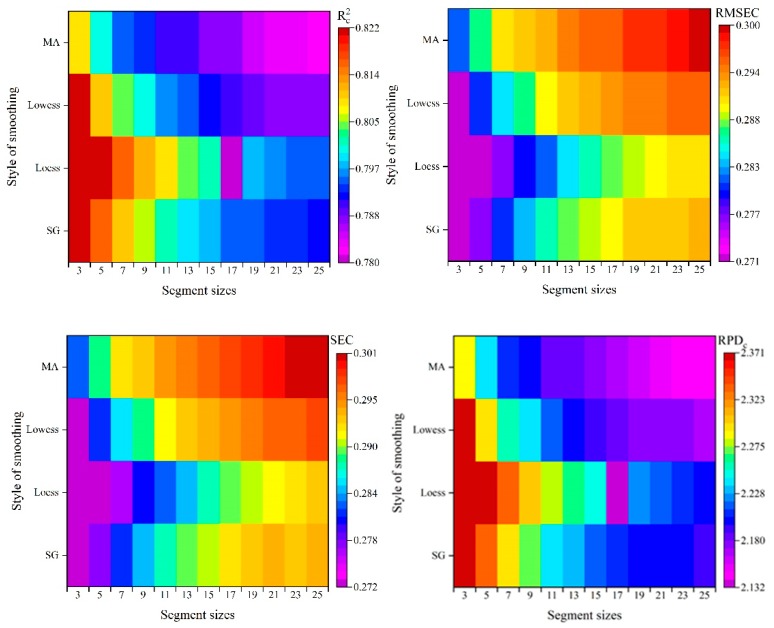
Results of PLS model for various segment sizes. MA: moving average; SG: Savitzky-Golay.

After the optimal parameters were obtained, moving average, loess, Savitzky-Golay, and lowess were used for further de-noising analysis. The selection of an appropriate segment size parameter is a key point. Figure 5 shows the de-noising effects of different segment size for moving average, loess, Savitzky-Golay, and lowess.

As seen in Figure 5, $R_{c}^{2}$ and ${\mathrm{RPD}}_{c}$ decreased gradually when segment size increased from 3 to 25 for the four pretreatment methods, while RMSEC and SEC increased with increase of segment size. For pretreatments, the best performance was obtained with three segment size. Except for moving average ($R_{c}^{2}=0.809$), the $R_{c}^{2}$ of the best model was the same ($R_{c}^{2}=0.822$). The performance of the model with 25 segment size was the worst, while loess obtained the worst performance with the segment size of 25. However, the $R_{c}^{2}$ of the worst models were all larger than 0.78.

### 3.5. Establishment of Vis-NIR Models for Wood Tracheid Length

The spectral data pretreated by LWT, LCM algorithm, and wavelet transform (db2 with fourth level) were then modeled by PLS. In terms of the results (Table 4), compared to the raw model, the modeling results pretreated by LWT-LCM and WT were inferior to. However, the performances of calibration and validation models were improved after the LWT pretreatment. As for the calibration model, the $R_{c}^{2}$ and ${\mathrm{RPD}}_{c}$ were improved by 2% and 4%, respectively, while RMSEC and SEC were decreased by 3% and 3%, respectively. Additionally, the performance of LWT was better than loess, Savitzky-Golay, and lowess.

**Figure 6:**
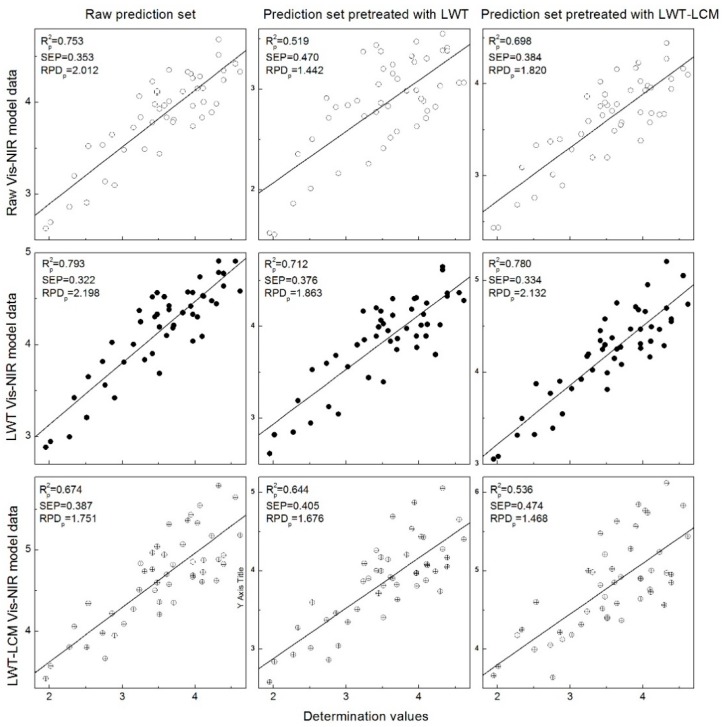
Predicted versus measured wood tracheid length for various prediction sets.

Even though WT had the same $R_{c}^{2}$ as LWT-LCM, the spectra of LWT-LCM model were constructed by the absorbance of the decomposition level with the largest r. Therefore, the prediction accuracies of the other three models for prediction sets with various pretreatments were analyzed. As shown in Figure 6, regardless of various preprocessing methods for the prediction sets, the model whose spectral data were processed by LWT exhibited a good fit for three prediction sets with an $R_{p}^{2}$ larger than 0.700. However, the performance of LWT-LCM coupled model was lower, which may be attributed to more error in the calibration model. When different prediction sets were used, the following three models obtained better results in untreated prediction set than other prediction sets. For the LWT-LCM coupled model, the $R_{p}^{2}$ was higher for prediction set with LWT preprocessing than that for LWT-LCM preprocessing. However, the situation was reversed for the raw model and LWT model.

## 4. Discussion

This study demonstrated that the tracheid length had a positive linear correlation with annual ring, which is consistent with the results of Boruszewski et al. [28]. For different pretreatment methods, the spectral data pretreated with the optimal LWT de-noising parameters (db2 wavelet with the fourth decomposition level under the global fixed hard threshold) can be used to better remove noise and extract characteristic signal, and thus obtain better prediction results for tracheid length ($R_{c}^{2}$ = 0.834) than raw model ($R_{c}^{2}$ = 0.822). With comparisons of previous studies on the prediction of tracheid length based on NIR technology, the accuracy of model in this study is inferior to the results in Schimleck et al. [29] ($R_{c}^{2}$ = 0.88), even though the samples are the same (*Pinus*), different geography and environment could induce different results. Additionally, different wavelengths for calibration were used. For the prediction of tracheid length in other species, although the accuracy of raw model in this study is a little lower than the results of Norway spruce [30] (0.828), the results of LWT pretreatment is better than spruce, which demonstrated that the feasibility of de-noising based on LWT.

The ability to effectively predict tracheid length was due to the change of Vis-NIR spectra with annual ring and there was a significant correlation between ring, chemical properties (lignin and cellulose as associated with 1415, 1965, and 2315 nm), and tracheid length [31].

However, after LWT coupled with the LCM algorithm pretreatment, model accuracy was lower than the raw model, and Figure 6 shows that the $R_{p}^{2}$ of the LWT-LCM model was lower than others (excluding the prediction set with LWT). This could be caused by the unnecessary wavelengths embedded in the reorganized data matrix which decrease the correlation in the characteristic band and results in more prediction errors. To further verify this speculation, correlation analysis between tracheid length and corresponding pretreatment spectra were studied.

**Figure 7:**
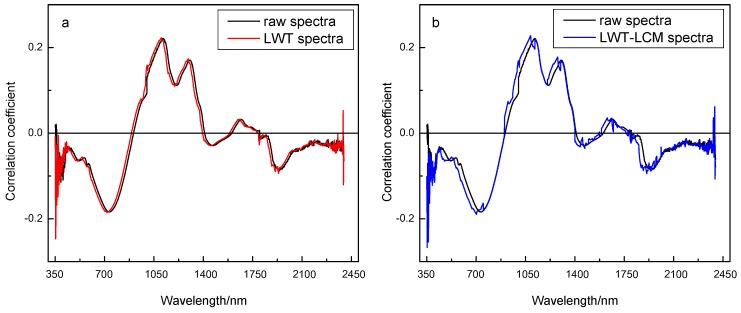
Correlation between corresponding spectra and tracheid length values. (**a**) The comparison of correlation coefficient between raw spectra and spectra pretreated by LWT; (**b**) the comparison of correlation coefficient between raw spectra and spectra pretreated by LWT-LCM.

As shown in Figure 7a, there were four main peaks (maximum value of correlation coefficient) at around 438 nm, 1114 nm, 1306 nm, and 1670 nm, respectively, and four main valleys (minimum value of correlation coefficient) were present near 734 nm, 1212 nm, 1470 nm, and 1944 nm, respectively. The correlation coefficient curves for raw spectra and spectra pretreated by LWT exhibited a uniform pattern, and there was little change compared to that of the raw spectra. However, in terms of the results for spectra pretreated by the LWT-LCM algorithm (Figure 7b), in the range of 1409–2397 nm, the unstable correlation coefficient results in a large fluctuation. Additionally, in the wavelengths of peaks and valleys, the correlation of spectra pretreated by the LWT-LCM algorithm was slightly lower than that of the raw spectra. This indicates that the speculation is reasonable.

## 5. Conclusions

The tracheid length values were generally high and exhibited a strong positive linear correlation with annual rings (ring from pith). The modeling results with different de-noising methods were different. The optimal parameters for larch wood spectra were db2 wavelet with decomposition layer four based on the lifting scheme. The LWT model with optimal de-noising parameters had relatively better accuracy and precision. However, the LWT-LCM model was not a good fit, which may be due to the unnecessary wavelengths embedded in the reorganized data matrix that decrease the correlation in characteristic bands and result in more prediction errors. Regarding models with different pretreatments, better prediction results were obtained for prediction sets with raw spectra than those of other sets. This study mainly discussed the de-noising of wood Vis-NIR spectra for improving the prediction accuracy of wood tracheid length based on different de-noising methods. In a follow-up study, more applications for prediction of wood’s properties based on smoothed data will be explored to provide a theoretical basis and technical support for the optimization of wood Vis-NIR spectra.

## Figures and Tables

**Table 1:** Summary statistics on tracheid length of larch in each data set.

Sample Set	No. of Samples	Max (mm)	Min (mm)	Avg. (mm)	SD (mm)	Skewness	Kurtosis
Calibration Set	117	4.169	1.626	3.234	0.645	−0.715	−0.539
Prediction Set	47	4.618	1.951	3.534	0.678	−0.614	−0.285
Total	164	4.618	1.626	3.320	0.667	−0.603	−0.421

**Table 2:** Results of PLS model of various wavelet with fifth decomposition level.

Wavelet	PCs	$R_{c}^{2}$	RMSEC	MAPE_c_ (%)
sym5	7	0.783	0.300	8.185
bior5.5	5	0.395	0.500	13.460
rbio5.5	7	0.503	0.453	12.407
db5	7	0.811	0.279	7.639

**Table 3:** Results of PLS model of dbN wavelet family with fifth decomposition level.

dbN	$R_{c}^{2}$	RMSEC	MAPE_c_ (%)
db1	0.807	0.282	7.782
db2	0.818	0.274	7.443
db3	0.763	0.313	8.738
db4	0.809	0.281	7.839
db5	0.811	0.279	7.639
db6	0.600	0.406	10.971
db7	0.789	0.295	8.304
db8	0.444	0.479	13.384

**Table 4:** Model statistics of wood tracheid length. Raw: raw model; LWT: model with LWT pretreatment; LWT-LCM: model with LWT coupled with LCM pretreatment; WT: model with WT pretreatment.

Model	PCs	Calibration Set				Validation Set			
		$R_{c}^{2}$	RMSEC	SEC	${\mathrm{RPD}}_{c}$	$R_{p}^{2}$	RMSEP	SEP	${\mathrm{RPD}}_{p}$
Raw	7	0.822	0.271	0.272	2.370	0.714	0.347	0.349	1.870
LWT	7	0.834	0.262	0.263	2.454	0.722	0.344	0.345	1.897
LWT-LCM	7	0.816	0.276	0.277	2.331	0.683	0.365	0.367	1.776
WT	7	0.816	0.275	0.277	2.331	0.717	0.347	0.346	1.880
